# What role can unmanned aerial vehicles play in emergency response in the Arctic: A case study from Canada

**DOI:** 10.1371/journal.pone.0205299

**Published:** 2018-12-18

**Authors:** Dylan G. Clark, James D. Ford, Taha Tabish

**Affiliations:** 1 Department of Geography, McGill University, Montreal, Canada; 2 Priestley International Centre for Climate, University of Leeds, Leeds, United Kingdom; 3 Qaujigiartiit Health Research Centre, Iqaluit, Canada; University of Mississippi Medical Center, UNITED STATES

## Abstract

This paper examines search and rescue and backcountry medical response constraints in the Canadian Arctic and potential for unmanned aerial vehicles (UAV) to aid in response and preparedness. Semi-structured interviews (n = 18) were conducted with search and rescue responders, Elders, and emergency management officials to collect data on current emergency response and potential for UAV use. UAV test flights (n = 17) were undertaken with community members. We analyzed five years of weather data to examine UAV flight suitability. Numerous challenges face Arctic search and rescue and backcountry emergency response. Changing social and environmental conditions were described as increasing vulnerability to backcountry emergencies. Responders desired additional first aid and emergency training. Legal and weather restrictions were found to limit where, when and who could fly UAVs. UAVs were demonstrated to have potential benefits for hazard monitoring but not for SAR or medical response due to legal restrictions, weather margins, and local capacity. We find that communities are ill-prepared for ongoing SAR demands, let alone a larger disaster. There are numerous limitations to the use of consumer UAVs by Arctic communities. Prevention of backcountry medical emergencies, building resilience to disasters, and first responder training should be prioritized over introducing UAVs to the response system.

## 1. Introduction

**Fig 1:**
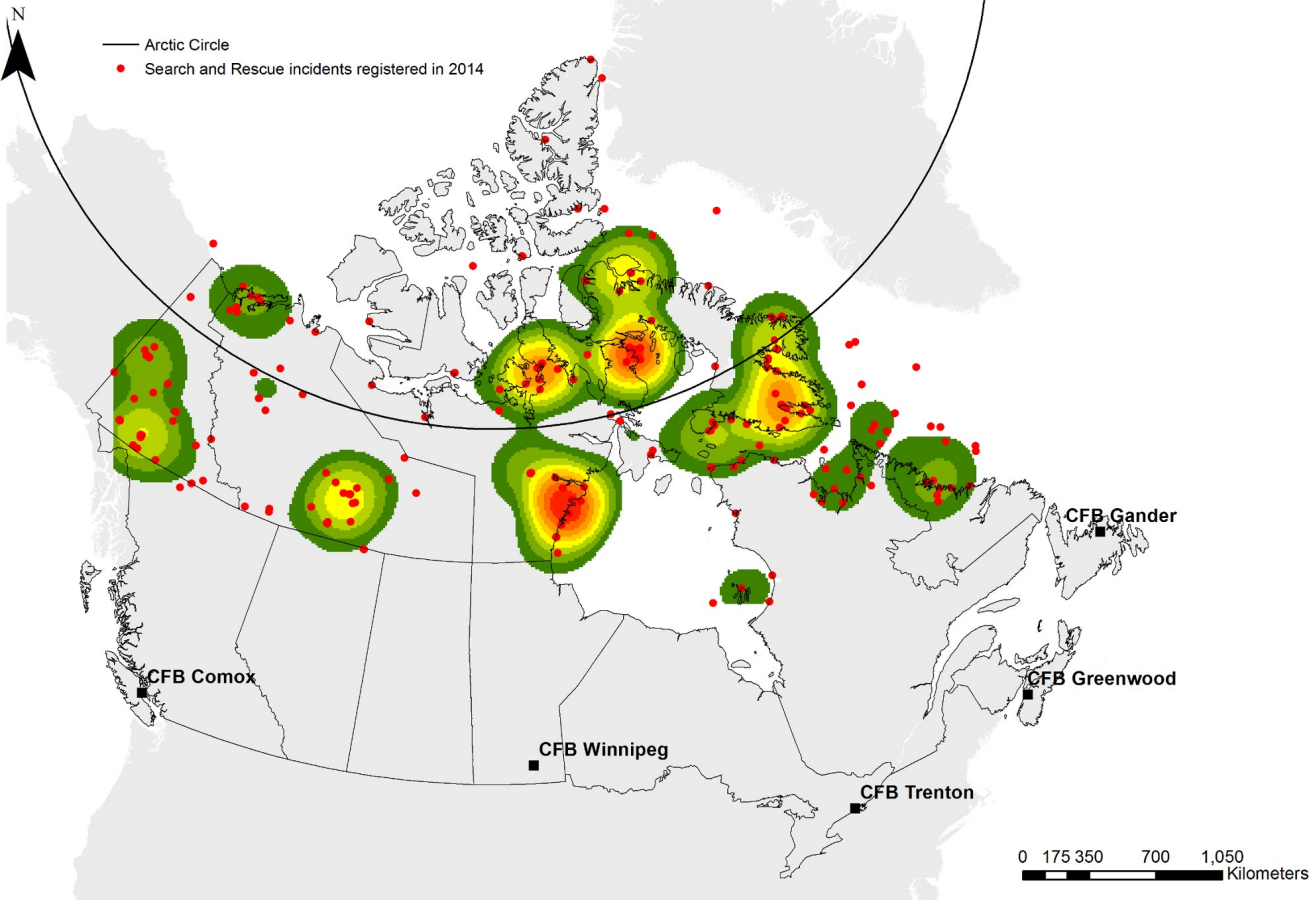
Canadian Arctic search and rescues. In 2014, there were 543 SAR incidents above 55^o^N. This map depicts the hot spots of incident location. Data from National Search and Rescue Secretariat. Basemap shapefiles are modified and republished from Government of Canada under a CC BY license, with permission from Natural Resources Canada and Crown-Indigenous Relations, original copyright 2018. Contains information licensed under the Open Government License–Canada [11,12].

The Canadian Arctic is experiencing transformative change. Over the past 30 years, temperatures have increased by 1.9°C–a rate more than double the global average–influencing permafrost, sea ice patterns, and weather extremes [1]. Paralleling rapid environmental change, demographic and economic transitions and socio-cultural changes are altering community development [2–4]. Culminating effects of compounding changes range from mental health stresses to increasing disaster risk across the region [5–8]. Rates of search and rescue (SAR) and backcountry injuries, for example, have more than doubled over the past decade, with an estimated rate of 7.81:1000 individuals requiring SAR above 55^o^N in 2014–16.4 times the national average (Fig 1) [6]. Further, increasing maritime traffic has contributed to concern over a cruise ship emergency in the region [6, 9]. Regional physical changes are altering the projected frequency and severity of storm surges, weather related infrastructure damage, and disruptions to community food and water systems, with resulting emergent and long-term health implications [7, 8, 10].

Addressing these emerging health risks is a priority across all levels of government in northern Canada and across the circumpolar north. Policies are increasingly promoting community-based interventions which attempt to confront colonial top-down decision making and are rooted in Indigenous knowledge systems, agency, and lived experience [13]. Given the physical isolation of most Arctic communities, it is also essential that resources and capacity are strong at the community scale. Community monitoring projects developed over the last decade include tracking mental health, subsistence harvesting, and infectious diseases [14]. Similarly, health promotion and climate change adaptation are regularly conducted locally. Disaster and mass-casualty emergency response capacity, however, remain centred at regional and federal levels, despite reliance on communities for day-to-day emergency response [15]. This creates a disconnect between local and federal emergency resource allocations, negatively affecting community health.

Community-based monitoring projects and health interventions in Arctic Canada are beginning to capitalize on new digital technologies, such as remote presence medical devices, iPads, sonar sensors, and smartphone apps to bridge north-south resource and capacity gaps [16–18]. In line with this trend, there is growing interest in use of unmanned aerial vehicles (UAVs) for community supported health initiatives, mainly for SAR, emergency management, and environmental mapping.

Outside of the Arctic, UAVs have been successfully applied in disaster response, backcountry and remote medicine, hazard monitoring, and for capturing environmental hazards [19–22], while in the Arctic, medium-size (1kg– 25kg) drones with potential to fly beyond line of sight have been discussed as being potentially useful for monitoring of marine mammals, building situational awareness during emergencies and defense operations, and use during oil spills. The private sector has also begun to capitalize on potential for UAVs by producing aerial maps of coasts and communities, though implementation has been limited as compared to other regions [23–27]. Similarly, large UAVs that are capable of flight beyond line of sight and can carry additional payloads, such as infrared sensors or LIDAR, are being increasingly used by federal agencies in the United States and Canada to monitor ice cover and shipping route, maritime surveillance, and practicing integration into defense and maritime systems. [26, 28–30].

Despite increasing interest in health applications of UAVs, no study to our knowledge has examined the opportunities, challenges, or standards of practice for using UAVs for Arctic health applications (monitoring coastal hazards, delivery of communication devices or first aid supplies during emergencies, or search and rescue). This is a significant gap given the accessibility of consumer UAVs which now cost under $3000 and the emphasis on community capacity in northern heath policy and broader strategic defences priorities [6, 9]. In this context, we examine if UAVs can address capacity gaps of communities across the Canadian Arctic to respond to backcountry emergencies and local hazards. While the study focuses on Canada’s Inuit communities, the findings have broader application to remote Arctic settlements across the circumpolar north and in low-resource remote communities.

## 2. Background

**Fig 2:**
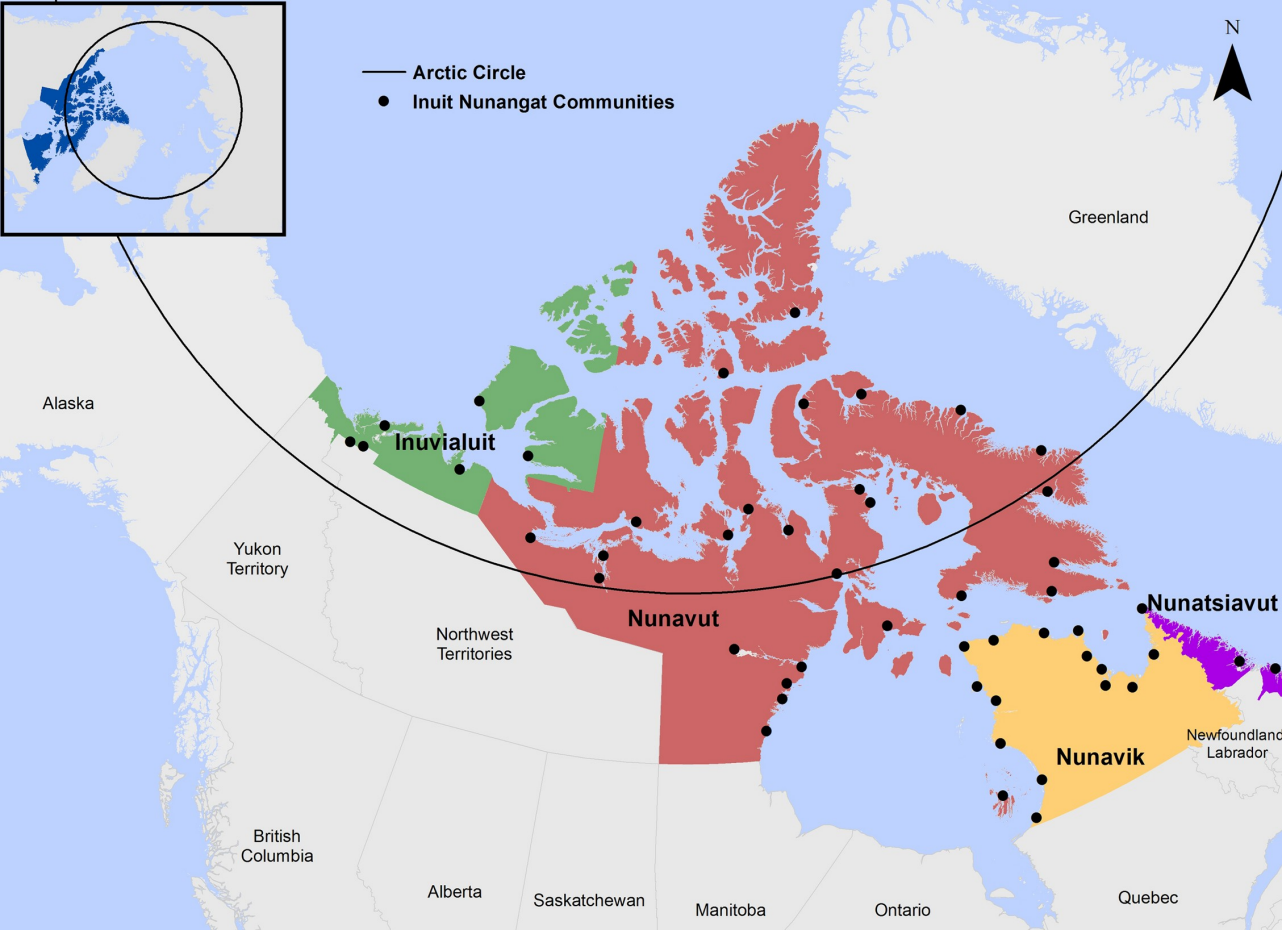
Inuit Nunangat. Encompassing roughly 4 million km^2^, the Inuit region of the Canadian Arctic–known as Inuit Nunangat–represents 35% of Canada’s landmass and over 50% of coastline [11,12]. Basemap shapefiles are modified and republished from Government of Canada under a CC BY license, with permission from Natural Resources Canada and Crown-Indigenous Relations, original copyright 2018. Contains information licensed under the Open Government License–Canada [11,12].

Encompassing roughly 4 million km^2^, the Inuit region of the Canadian Arctic–known as Inuit Nunangat–represents 35% of Canada’s landmass and over 50% of coastline (Fig 2). With approximately 54,000 people spread over 53 communities, quality healthcare delivery, connectivity, and community services are challenged by extreme remoteness across the Canadian Arctic. Traditional subsistence activities and harvesting remain central to Inuit cultural identity, food security, and wellbeing, and have been linked to a range of positive health outcomes and are one of the central focuses of regional health initiatives [31]. These hunting, fishing, and foraging activities, are generally referred to as ‘being on the land’. Travel between communities by snowmobile, all-terrain vehicles (ATV), and boat to visit family members, purchase supplies at a regional hub is common in the Canadian Arctic, with distances ranging from 50km to 300km [5, 32–34].

Travelling on the land in the Arctic is inherently dangerous, with Indigenous knowledge evolving to manage these dangers. Indigenous knowledge refers to a “cumulative body of knowledge, practice and belief, evolving by adaptive processes and handed down through generations by cultural transmission, about the relationship of living beings (including humans) with one another and with their environment,” and for Inuit includes factual knowledge of how to safely use the environment (e.g. weather and ice forecasting and survival skills) and culturally based value statements and cosmology from which explanation and guidance is derived [35]. Elders describe acquiring Indigenous knowledge to be a lifelong process that involves continuous observation and learning when on the land and listening to others.

Individuals across Arctic Canada report that hazards and weather are increasingly difficult to read and predict due to social and environmental changes [32]. It is common for experienced land-users to use all resources available to them before traveling on the land, this includes talking to others who have been out recently on CB radio, checking weather forecasts online–when internet is functioning–and using Indigenous knowledge [34]. Hazard monitoring is also being used in some community-based projects as a means of further supplementing traditional observation with information about coastal processes, stream levels or ice conditions [17].

**Fig 3:**
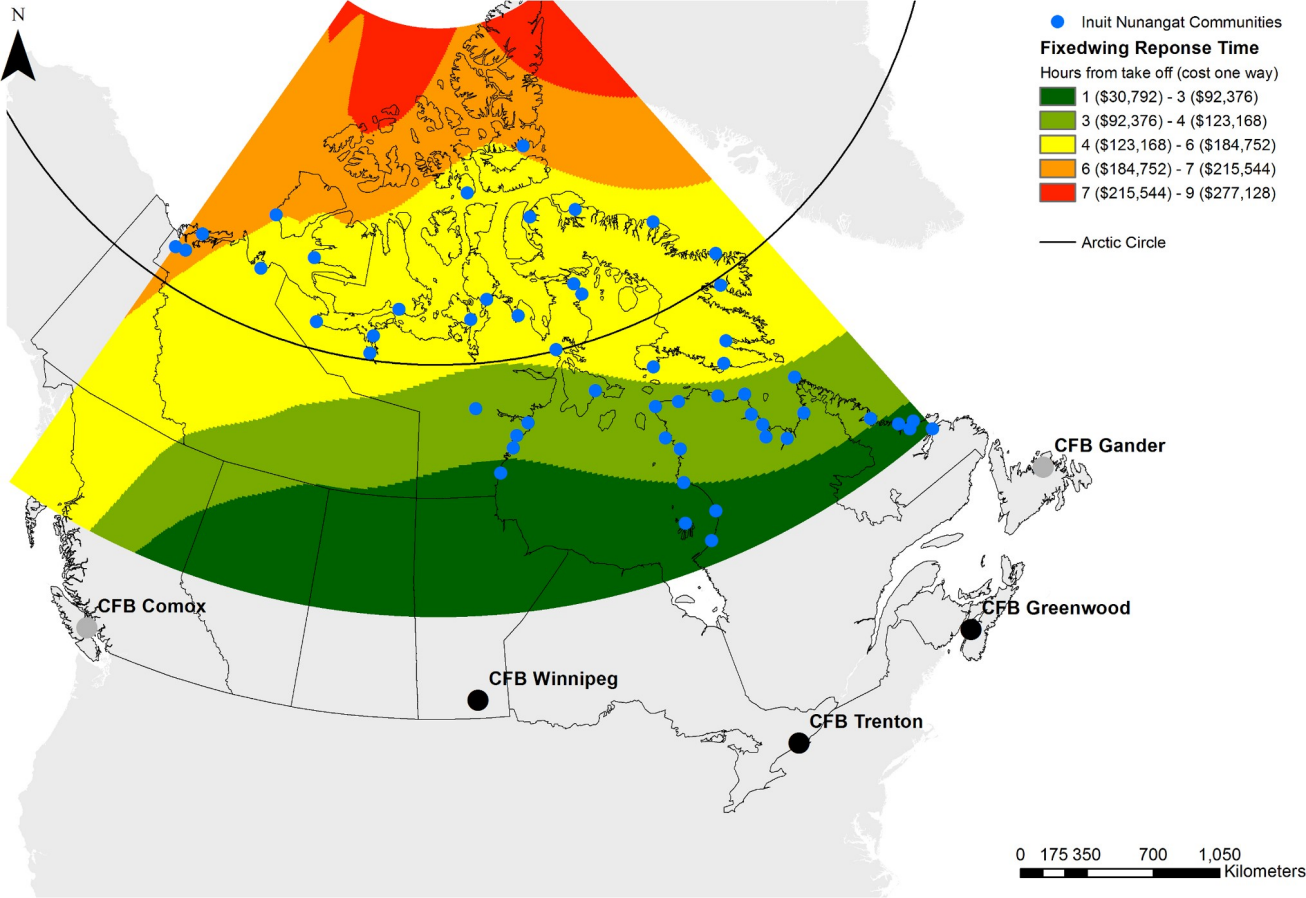
Royal Canadian Air Force Arctic response times and cost. Travel times for RCAF CC-130 SAR assets from takeoff to location are depicted above. RCAF has CC-130 currently at CFB Winnipeg, CFB Trenton, and CFB Greenwood. The map shows time from the closest aircraft, if aircraft were only dispatched for their zone, times in the far north would increase by roughly 2 hours. More information about calculations, as well as times for helicopter responses, can be found in the S1 File. Basemap shapefiles are modified and republished from Government of Canada under a CC BY license, with permission from Natural Resources Canada and Crown-Indigenous Relations, original copyright 2018. Contains information licensed under the Open Government License–Canada [11,12].

Emergency medical response and SAR in the vast majority of communities in the Canadian Arctic is largely reliant on groups of volunteers in each community with no local prehospital emergency medical services. While most SAR incidents do not involve the Canadian Coast Guard (CCG) or Royal Canadian Air Force (RCAF), when they are required, RCAF aircraft generally have to travel long distances (Fig 3, S1 and S2 Fig). Similarly, while the CCG has a presence across the Arctic during the shipping season, they are seldom used for SAR incidents year-round, though Coast Guard Auxiliary volunteer units in a number of communities are increasingly being relied on.

## 3. Methods

In this study, we drew from community-based research frameworks that are widely applied in across the Canadian Arctic and in human dimensions of climate change literature [36–38]. Specifically, the semi-structured interviews, triangulation of mixed data sources, and integrated data collection and analysis were rooted in grounded theory and post-positivist perspectives [36, 39, 40].

To investigate if UAVs can address capacity gaps of communities to respond to backcountry emergencies and local hazards, we use a case study from the community of Arviat, Nunavut. Focusing on one community allows us to take an in-depth examination of the opportunities and challenges of using UAVs, while developing an understanding of the complexity behind SAR and emergency response in the Arctic (S1 File). Selection of Arviat reflects the fact that the flat landscape is conducive to UAV use, a variety of land types (land, water, and sea ice) are present near the community, and the local SAR group and hamlet was eager to participate in the research project. Arviat is located on the western shore of the Hudson Bay, with population of 2657 (93% Inuit). The settlement has a primary health centre with 4–5 nurses and physicians that fly in throughout the month. Between 2014 and 2017, there were an average of 30 official searches per year in Arviat, the majority related to mechanical breakdowns.

The project was co-developed with the community and guided by principles of community based participatory research in order to increase community buy-in and ensure that the project had some benefited to Arviat and the region [13]. Project conceptualization came largely from discussions with community members and were rooted in their interest in using UAVs for monitoring wildlife and search and rescue. Building on already strong researcher-community relationships, we worked closely with hamlet officials, search and rescue volunteers, youth leaders, and expert harvesters to develop the study objective, methods, and deliverables. There was ongoing feedback and adjustments to the study objectives throughout the time period, including reducing the number of on the land trainings to respect volunteer time commitments and ensuring a UAV was left in the community for further use. Study questions were also rooted in the arctic human security, emergency management, and remote sensing literature that highlight knowledge and resource gaps around regional search and rescue capacity [9, 41–43] and call for exploration of UAVs in arctic emergency management [23, 28, 29, 44, 45].

**Fig 4:**
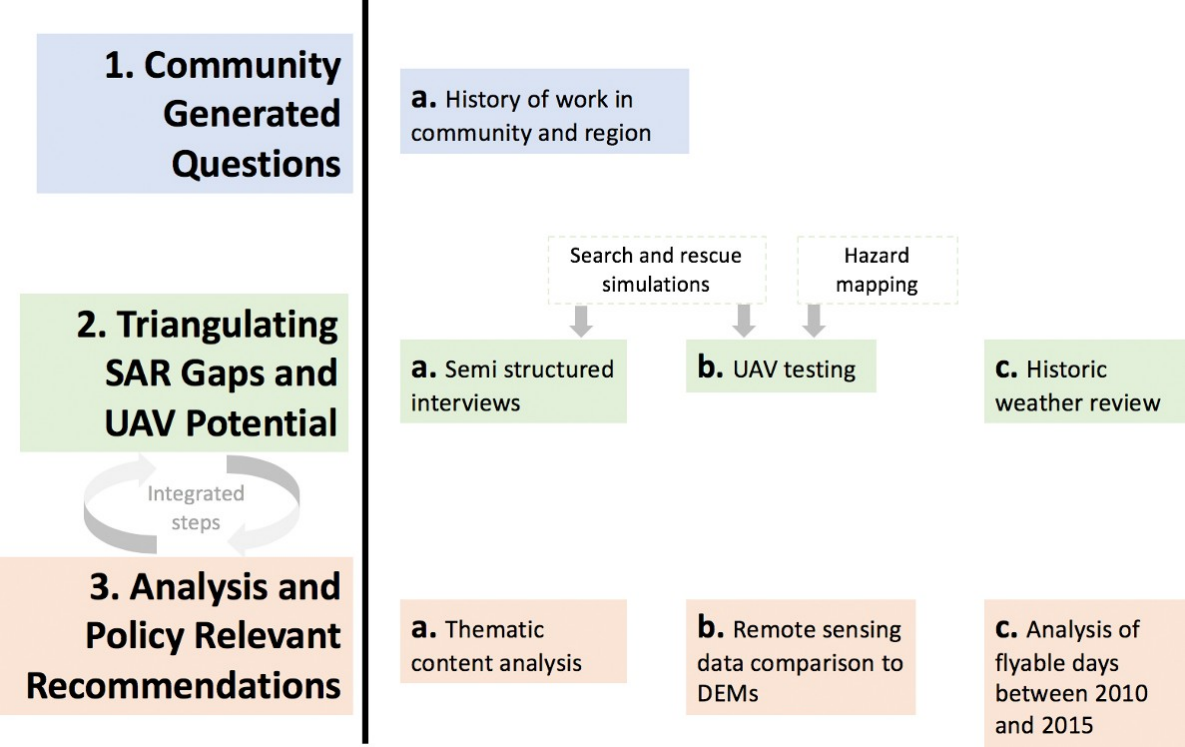
Study methodological process. The study draws from community-based research methods. Questions and the conceptualization of the study were generated by community interests. Data were gathered from qualitative interviews, participatory testing of UAVs, and Environment Canada historic weather observations. Drawing from grounded theory and common methods applied in the region, analysis was iteratively integrated into data collection.

We combine qualitative and quantitative methods to assess: 1) how UAVs could address identified SAR and disasters capacity challenges; 2) potential for small and medium size UAVs to be legally and safely flown by community governments or volunteer groups; 3) if UAVs can be flown in Arctic conditions and how this varies over the year; and 4) conceivable UAV uses for SAR, hazard mapping, and additional emergency management tasks (S1 File). These study questions about the current SAR context and UAV capabilities led to the development of three areas of analysis (a. semi-structured interviews; b. UAV testing; c. historic weather review) (Fig 4)

Semi-structured interviews were conducted with SAR volunteers (n = 7), harvesters (n = 6), Elders (n = 3), and individuals involved in emergency management from the community and region (n = 5), with some individuals representing multiple roles. The average estimate age of participants was 46, with 3 female participants and 15 males (S1 File). Questions focused on emergency/SAR burdens in the community, response practices, use of electronic technology on the land, and wayfinding and observation of environmental conditions and UAV use. This study was conducted under human subject ethics guidelines and research licenses. A research license for this study was approved by McGill University and Nunavut Research Board.

UAV testing was conducted on 17 occasions between March 2016 and June 2017 to observe how useful UAVs could be for spotting ground targets, ease of operation in the region, and potential for use with hazard monitoring (S1 File). Live testing created avenues for greater community participation in the research and allowed us to verify constraints and opportunities. Flight dates were selected to capture winter, spring, and summer conditions using a DJI Phantom 3 and DJI Phantom 4 and iPhones for UAV video feeds and image capturing. These UAV models were selected due to their low cost to high maneuverability and imaging capabilities; while models that can fly in harsher conditions and that have inferred imaging are available, these models are very cost prohibitive. During test flights, participants were asked to first fly above nearby landmarks, and then find targets set up on the land (tarps). Targets used were meant to simulate a tent or hunting party. Seven flight sessions were done with community participants and ten additional flights conducted by research team. A total of 19 individuals were involved as participants or observers.

Hazard mapping was conducted by the research team at various locations near Arviat, selected to capture a mixture of ice, snow, water, and land and were over 9km from the Arviat airport. Flights were conducted at 23m and 38m above ground level (AGL). All hazard mapping was conducted with the DJI Phantom 4 and by capturing 12.4 mega pixel JPEG images (S3 and S4 Fig). Accurate and near-real time orthostatic maps and DEMs have potential to aid in monitoring of coastal changes, ice conditions, stream levels, and fire patterns, aiding emergency planning for SAR and disasters by improving situational awareness.

To examine how conducive the weather conditions across the Canadian Arctic would be to consumer available UAVs, we reviewed wind speed, visibility, and temperature conditions in Arviat for each day from 2010 to 2015 from Environment Canada daily and hourly observations (S1 File). Out of the 2192 daily observations between 2010 and 2015, there were 384 days with missing visibility observations, 26 days missing with max wind speeds, and 18 days missing minimum temperature. Days with missing values were deemed non-flyable, and no temporal pattern to missing observations was noted. To quantify the number of potential days that were flyable using a consumer UAV–likely the only affordable option for most communities–we established two categories of minimum conditions limits (Table 1).

**Table 1:** UAV flight weather limitations. In order to quantify how many days annually a consumer UAV could be flow in Arviat, Nunavut, we established two categories of minimum parameters and assessed 5 years of daily weather data. Limits were based on manufacture recommendations, legal limits, and tests conducted in Arviat.

Scenario	Min Daily Temp	Avg Daily Wind Speed	Max Daily Wind Gust	Visibility Min
High margin cut off	> -5 ^o^C	< 20km/hr	< 40km/hr	> 1km
Medium margin cut off	> 0 ^o^C	< 15km/hr	< 35km/hr	> 2km

Analysis of interviews followed an iterative approach outlined in our previous studies and widely applied in the field. This included thematic content analysis and the following iterative steps: 1) Data within each thematic category were reviewed for primary themes; 2) interviews were re-listened to, noting conversational tone; 3) data themes were mapped out within categories and links drawn (S1 File) [31, 46]. We use quotes from interview to illustrate with participants’ own words perspective on SAR and UAV use. Interviews were analysed throughout the research process, allowing the research team to further explore emerging themes with UAV testing and additional interviews [39].

Hazard maps were analyzed by processing images with Pixel4D, then assessing output GeoTIFF files in QGIS. Orthostatic maps were compared to available satellite images to assess processing alignment, detail, and coverage. UAV generated DEMs were compared to DEMS available through Natural Resources Canada, and 3D mesh outputs were compared to one another and to the research team’s knowledge of the landscape to determine quality.

## 4. Results

### 4.1 Perspectives and challenges facing search and rescue

In Arviat, the SAR committee has twelve active members ranging in age from young adults (over 18) to Elders. Many volunteers reported a lack of training and resources needed to respond to local incidents. Roughly 2/3 of volunteers reported not having received any first aid training in the past five years, with many members reporting anxiety around potentially dealing with medical conditions perceived as complex (e.g. anaphylaxis or cardiovascular compromise). Volunteers expressed confidence in dealing with more common injuries, such as hypothermia or frost bite (S1 File). With increasing SAR incidents related to illicit substance trafficking, participants also expressed uncertainty about how to deal with aggressive individuals. As one individual stated: “[I am concerned about] our safety, mostly when [the missing party’s family] says ‘we don’t want the [police] involved’. What about if we get beat up …? They are always threatening [us]”. Volunteers also expressed the emotional toll of responding to SAR incidents in the context of small communities where everyone knows one another, contributing to burnout and high turnover rates.

Participant reported strengths of the SAR volunteers included strong knowledge of the land and local hazards, survival knowledge and skills, and adaptability to new technologies (using satellite beacons and phones and GPSs). While some participants, especially Elders, preferred traditional approaches (e.g. wayfinding using landmarks and snowdrifts) over use of electronic devices, the majority of participants expressed the importance of having a strong understanding of the environment rooted in Indigenous knowledge and also having electronic devices as a safety measure. As one SAR volunteer stated, “What Elders always say is don't just rely on those [electronics]… They're kind of worried that people are going to stop understanding the normal ways of navigating and those things because they have these devices. But then batteries die, and they are screwed.”

Another strength of the SAR group expressed is their ability to respond quickly to incidents and their knowledge of what is going on in the community. A participant noted “We can talk about technology, but [of] huge importance is listening to where people are going, being on CB, and communications [in Inuktitut]. Any kind of clue you get can narrow something down.” Indeed, RCMP officers reported often hearing about a missing person from SAR volunteers. Officers also noted their dependence on volunteers for incidents outside of town, with one officer noting: “To be honest … I have no business being out there. I am pretty savvy in the bush down south, but, this is different story … I am not qualified to be out there. I have only been here for three years and I don’t think you learn that over three years.”

In the context of capacity to respond to disasters, individuals involved in emergency management at community and regional levels expressed apprehensions ranging from preparedness for power outages to multi-casualty incidents. Noted vulnerabilities included medical capacity in the community, limited communication structures between organizations in communities and with regional offices, and a likely 8-hour or more wait for additional medical or SAR resources if required. Emergency management personnel also noted that in the Canadian Arctic a mass casualty incident could simply be an ATV rollover causing three life-threatening traumas–enough to overwhelm SAR, RCMP, and health clinic, and regional medical evacuation resources. Regarding medical capacity and dependence on flyable weather conditions a participant noted “[we] had an 18-year-old girl die waiting for an evac[uation]. [They]… died from an infection that shouldn’t have killed anyone.”

Discussing reliance on community power plants during, an emergency official stated “for lack of a better term, we’d be [obscenity] if the power went out. We are on oil furnaces, but they need electricity to run the furnaces. Minus 60 with the wind, the pipes would be frozen from 20 minutes to 30 mins.” Communications challenges discussed included health centre lines being tied up with community members calling to inquire about an incident, inability to call in additional staff quickly, and challenges quickly mobilizing SAR volunteer groups with no pager systems (which fire departments have).

Changing community demographics and weakening of land skills and knowledge among younger generations, were seen as additional factors affecting SAR and disaster management. As one emergency management official stated, “We have some very skilled very experienced locals that could survive with what they could fit on a [sled] out there for a long time. But, we're getting a lot larger percentage of our population that could not function in that situation for a day.”

### 4.2 Community application of UAVs for SAR and hazard recognition

**Fig 5:**
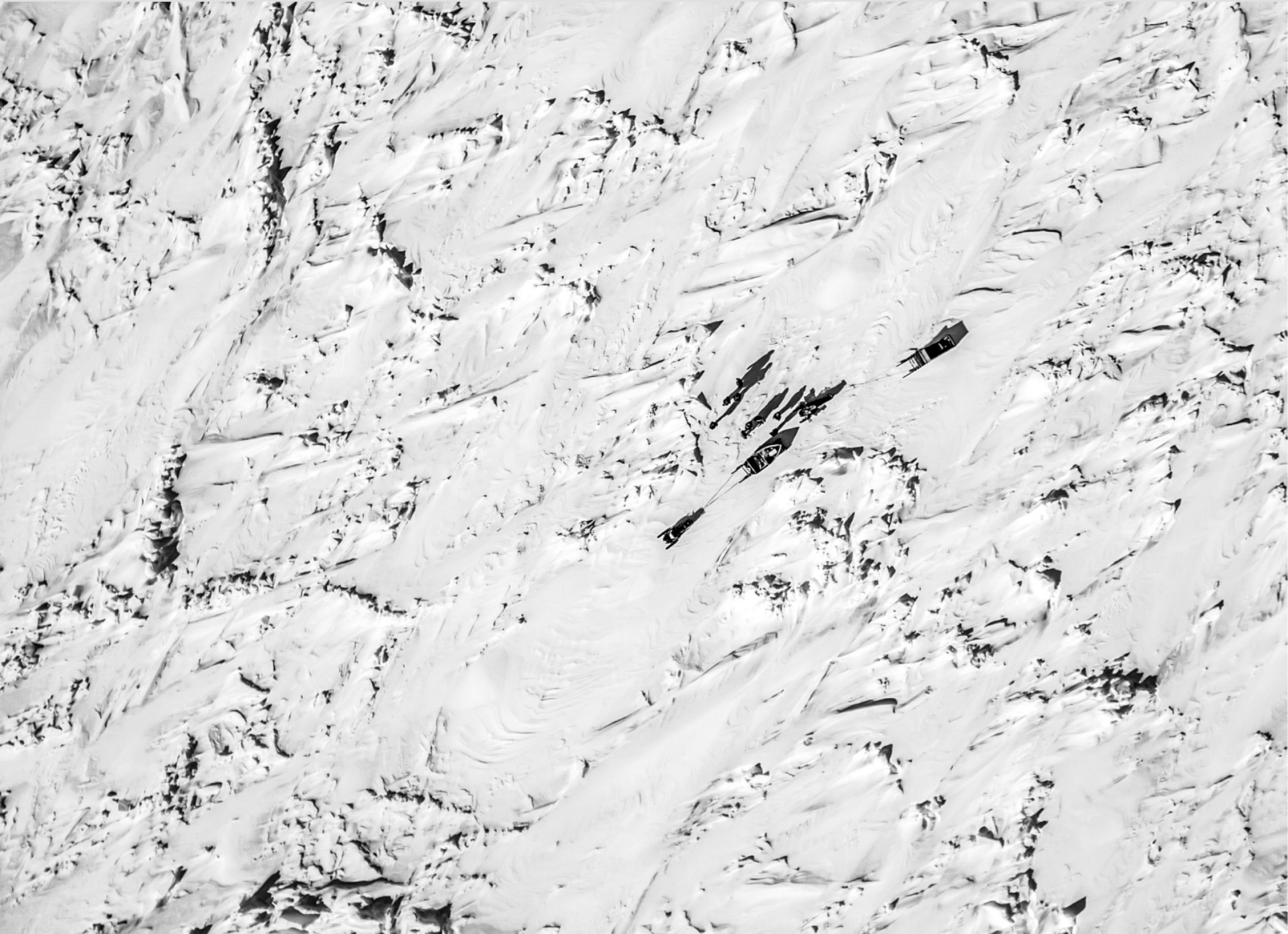
Headed to the floe edge. This photo, captured at roughly 50’ above ground level, shows a group traveling from Arviat, NU to the floe edge on the Hudson Bay. While this target is easy to see with a still image, participants found it difficult to see on an iPhone screen. Intense glare from the sun and snow reflection, along with cold conditions causing loss of hand dexterity made it very difficult to search for objects during these conditions. Photo taken by Dylan G. Clark, with approval to distribute under the CC BY license.

Three tests of UAVs were conducted during the last week of March, two tests were done in May, and two were done in June. Flying at 6m AGL, 20% of users were able to spot targets (1m x 2m tarp) after 10 minutes of flight, although the majority did not spot the target; targets were placed within 30m of UAV takeoff site. Users reported that even when they knew where the tarp was, it was difficult to spot when flying at 23m and above. While no user reported operating the UAV as being difficult, younger participants (under 25) grew comfortable quicker. Some of the youth commented that video game playing had made it easier to learn to fly the UAV. There were challenges in using iPhones as a primary screen for video feeds from the UAV. During colder conditions, iPhones often had critical battery levels long before other UAV system batteries failed, and screens were too small for adequate real-time spotting. Participants of all ages commented that it would be easier to fly and see the landscape with larger screens (Fig 5).

Participants expressed that UAVs would be particularly useful for searching over water and in areas with dense vegetation. One participant commented, “[Y]ou know, when I take it up I [see] lots of rocks in the water, even though it was 10 to 20 feet deep” [middle-age male harvester and SAR volunteer]. Individuals were also impressed by the speed at which the UAV could fly, covering distances far faster than ATVs, snowmobiles or boats. There were also discussions between SAR volunteers about being able to drop communication devices to people stranded on thin ice and potential for monitoring caribou herds and hunting grounds with the UAV. Young adults who tried flying UAVs were quick to learn flight controls and aircraft dynamics. Some of the youth commented that video game playing had made it easier to learn to fly the UAV.

### 4.3 Hazard mapping

Three coastal regions were mapped, all located roughly 9km from Arviat. Orthostatic maps produced from flights flown at 23m AGL were approximately 15cm pixel quality, while images captured at 38m produced approximately a 25cm pixel quality maps. UAVs were able to capture an area of roughly 0.085km^2^ while flying at 23m in a track crawl pattern, 0.052km^2^ area coverage with a 22m cross-hatch pattern, and a 0.15km^2^ area while doing a track crawl at 38m. While elevation maps were skewed by floating ice, snow drifts, rocky terrain, the majority of control points were within 1m of elevation data available for the region. Three dimensional meshes produced from the coastal mapping regions varied in quality. While elevation maps were skewed by floating ice, snow drifts, rocky terrain, as well as flight level variation throughout flight, the majority of control points were within 1m of NRCAN DEM data available for the region. UAV produced DEM resolution was roughly 30cm, while the best resolution of publicly DEMs is only 5m.

**Fig 6:**
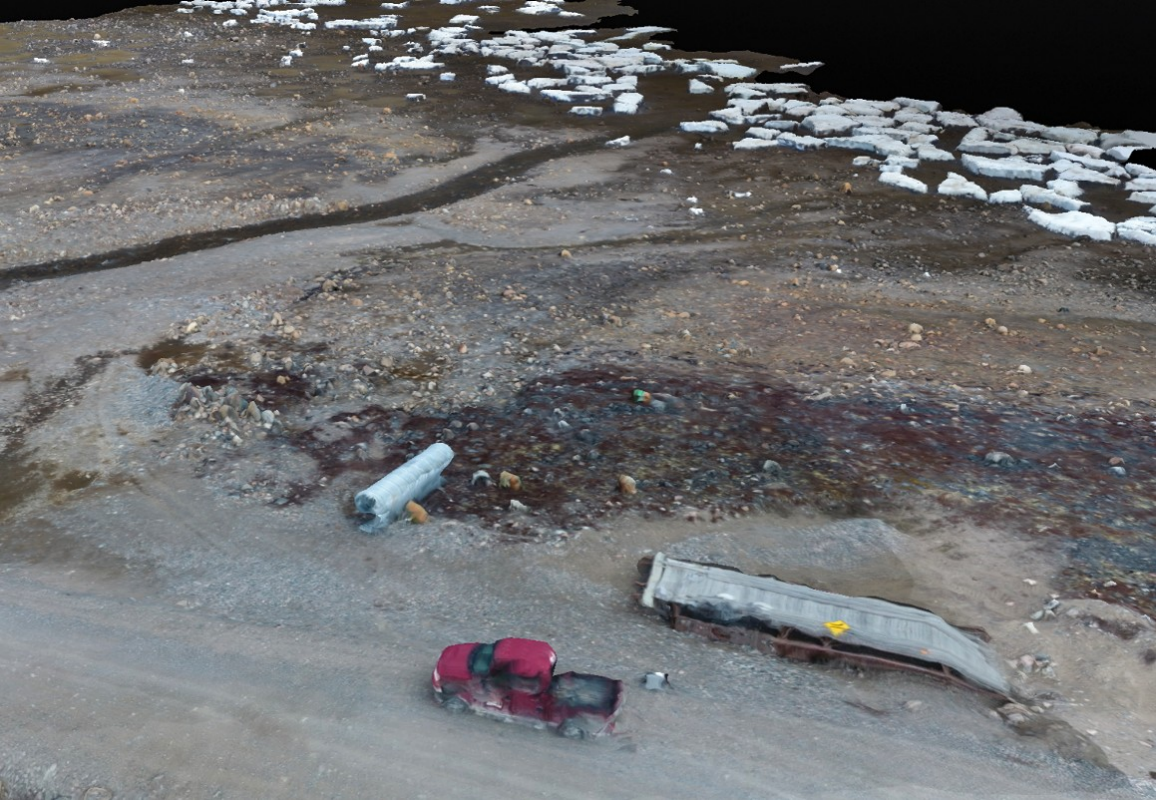
Three-dimensional rendering. This photo captures the power of the three-dimensional meshing produced using UAV images taken at approximately 75’ above ground level. Three-dimension meshing, processed by Pixel3D, produced near-real life textures that could be beneficial in hazard mapping and emergency preparedness. Photo generated by Dylan G. Clark, with approval to distribute under the CC BY license.

Three dimensional meshes produced from the three coastal mapping regions varied in quality (Fig 6). The highest quality 3D mesh layer was produced by the cross-hatch pattern flown at 23m. This 3D mesh clearly depicted vehicles, culverts, floating ice, and rocks larger than 60cm diameter. The second-best quality 3D mesh was produced with the track crawl flight pattern at 23m. This mesh depicted cabins, roads, and large snowbanks, though with less discernable quality. The 3D mesh quality from 38m was beneficial only for assessing large topographical features.

### 4.4 Legal limitations

While the legal regulations for medium UAVs (1g to 25kg) changed throughout our study, there were a number of broad limitations that are anticipated to continue to limit UAV capacity. Legal limitations include: inability to fly beyond the pilot’s line of sight, inability to carry droppable payloads, a maximum flight ceiling of 91m, can only fly in daylight when visibility is over 1.9km, and cannot fly in clouds. Further, research and commercial flights require flight training and liability insurance. Additionally, if you are flying within 9km of an aerodrome you must file additional documentation, requiring up to 3 months of prior notice. Given that an airport is within 9km of nearly every community in the Canadian Arctic, such requirements call for high levels of training and investment for communities to fly UAVs nearby town. It is beyond the scope of this study to examine the relevance of regulations to aviation safety in the unique setting.

### 4.5 Retrospective analysis of conditions for flight

**Fig 7:**
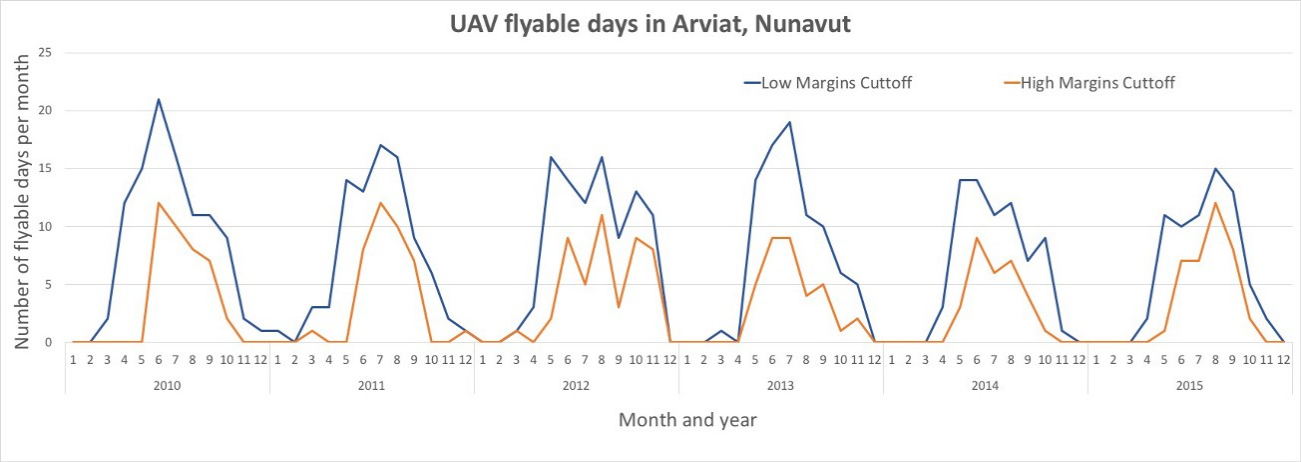
UAV weather constraints. Five years of daily weather observations were assessed for suitability of UAV flight. This graph depicts the number of days (under model 1 and 2) that were deemed suitable for UAV flights.

Under the high limit model, there were an average of 83.8 (95% confidence 72.5–95.2) flyable days per year. Under the medium limit model, there were an average of 38 (32.6–43.4) flyable days per year. The most flyable months under the high limits model were May, June, and July, averaging 17 flyable days per month (Fig 7). The most flyable months under the medium limits model were June, July and August, averaging 10.3 flyable days per month. The most common reason for a non-flyable day was due to wind speed (average of 236 fails/ year). Temperature (average of 192.8 fails/year) and visibility (average of 76.4 fails/year) were also limiting factors. It should be noted that the months with fewer good-weather-days for flying a UAV are been correlated with higher SAR rates [47].

## 5. Discussion

Arctic disasters and backcountry emergencies are likely to continue to change in frequency and severity over the coming decades, with the Arctic projected to see the most pronounced climate change globally [48]. Demographic transitions and changes in Indigenous knowledge systems have the potential to exacerbate these risks [35]. The benefits of community piloted UAVs during emergencies, however, are limited by legal restrictions, weather margins, and the inadequate number of individuals involved in emergency response in a community. There is, however, potential for communities to operate UAVs during planned mission in summer months, given that resulting mosaics and elevation maps are of high quality and can be regularly collected and used by communities to plan for emergencies and assess long-term public health effects of climate change. While more tests are needed to test UAVs for hazard mapping, it is likely that flight levels below 23m are required for DJI Phantom 4 or similar UAVs in order to gather high resolution images and elevation data.

### 5.1 Limitations and strengths

While the study led to an in-depth examination in the central Canadian Arctic, the generalizability of this case study is not completely known; extrapolation of study findings to other Arctic communities and regions demands further research. Additional research in other Arctic regions would be beneficial in developing an understanding of spatial variation of emergency preparedness and capacity to integrate emerging technologies. UAV legal regulations and UAV technology itself are continuing to change and should be monitored to assess feasibility shifts.

## 6. Conclusion

The central challenge for Arctic public health and disaster response is a geographic one, given the regions low population density and vastness. Historic approaches of the Canadian government have varied from forcibly relocating people, flying patients south for medical treatment, investing in federal capacities with hopes that fast mobilization could address local needs, or simply ignoring community-level needs and risks. None of these approaches to public health promote holistic community wellbeing.

Communities across the Arctic need to be ready to respond to an increasing number of SAR of both small and large scale. This requires capacity to monitor local hazards, deliver first response in disasters before federal assets arrive (12–24 hours), and integration of local, regional, national emergency management systems. We find that communities are largely ill-prepared for ongoing search and rescue demands, let alone a larger scale disaster, such as a cruise ship emergency.

Study findings have direct implications for public health practice and research. First, additional investments at the community level are needed to address prehospital emergency response gaps and meet federal expectations and reliance on communities to be first responders in local emergencies and larger disasters [5, 9, 33, 49]. Investments in wilderness first aid training and culturally-relevant courses on management of emergencies and of violent persons would likely be beneficial for improved delivery of care while reducing volunteer stress and burnout. Second, preparedness for larger scale disasters would likely improve with more frequent multi-agency exercises and if community volunteers had more exposure to incident command systems through integrated multi-agency training. Additional research is needed to better understand how Indigenous knowledge can be integrated into southern templates of emergency management. There is also need for improved structures for emergency management communication within communities, between agencies, and from communities to regional and federal scales, a designated structure for managing SAR resources at the community level, and greater funding for prevention. Finally, while emerging technologies have the capacity to reduce inequity of resources and capacity in communities across the Arctic and in other world regions, integration of technologies whether for community-based monitoring or healthcare delivery, need to be paired with systems of support, soft skill investments, and be adherent to cultural values.

## Supporting information

S1 File Supporting materials. The supporting materials file provides additional information about the context of search and rescue across the Canadian Arctic, study methods, and additional results.
(PDF)

S1 Fig RCAF helicopter Arctic response cost. Estimated response cost for CH-149 and CH-146 to respond to SAR incidents across the Canadian Arctic. Costs are estimated for the aircraft that could arrive at the location quickest from respective bases (CFB Comox, CFB Trenton, CFB Greenwood, and CFB Gander). Basemap shapefiles are modified and republished from Government of Canada under a CC BY license, with permission from Natural Resources Canada and Crown-Indigenous Relations, original copyright 2018. Contains information licensed under the Open Government License–Canada (11,12).
(TIF)

S2 Fig RCAF helicopter Arctic response time. Estimated response time from takeoff to incident location for CH-149 and CH-146 to respond to SAR incidents across the Canadian Arctic. Times are estimated for the aircraft that could arrive at the location quickest from respective bases (CFB Comox, CFB Trenton, CFB Greenwood, and CFB Gander). Basemap shapefiles are modified and republished from Government of Canada under a CC BY license, with permission from Natural Resources Canada and Crown-Indigenous Relations, original copyright 2018. Contains information licensed under the Open Government License–Canada [11,12].
(TIF)

S3 Fig UAV aerial image over the Hudson Bay. This photo captured at roughly 300’ above ground level, depicts two groups ice fishing on the Hudson Bay near Arviat Nunavut. In high contrast environments, such as melting sea ice, targets can be seen at higher altitudes with large screens. However, participants and researchers found it difficult to spot targets in lower contrast environments, such as bare ground, and while using smaller screens for real-time image feeds. Photo taken by Dylan G. Clark, with approval to distribute under the CC BY license.
(TIF)

S4 Fig Three-dimensional rendering. This photo captures the power of the three-dimension meshing produced using UAV images taken at approximately 75’ above ground level. In this photo you can see a wooden cabin, ATV trail, shore line dropping off to the right of the photo, and the visible sand and rock pits in the foreground. Photo generated by Dylan G. Clark, with approval to distribute under the CC BY license.
(TIF)
