# Supplementary material for: What role can unmanned aerial vehicles play in emergency response in the Arctic: A case study from Canada
Source: PLoS One. 2018 Dec 18;13(12):e0205299. doi: 10.1371/journal.pone.0205299 (PMC6298648; doi:10.1371/journal.pone.0205299)
Supplement: S1 File — The supporting materials file provides additional information about the context of search and rescue across the Canadian Arctic, study methods, and additional results. (PDF) [file pone.0205299.s001.pdf]

Supplemental Materials

1. Canadian Arctic and search and rescue background

Given the importance of harvesting and intracommunity travel to mental health, food security, identity, and socioeconomic wellbeing, communities and regional governments are interested in ensuring continued ability to safely engage in these activities. Search and rescue demand and backcountry emergencies, however, have been increasing dramatically over the past decade across the Canadian Arctic [1, 2]. Increasing SAR and backcountry injury burdens have been linked to a combination of factors, including less dependable snowmobiles and all-terrain vehicles that are more difficult to repair, diminishing knowledge of the land and survival, travelers and harvesters being in more of a rush as well as approaching being on the land differently, alcohol and illicit drug smuggling between communities, and increasing ease of calling for SAR through satellite beacons, cellphones, and satellite phones [3-5].

Climate change has also been observed as making it more difficult for harvesters and travelers on the land to identify hazards and increasing difficulty of travel [7, 8]. Though there are some cases of individuals falling through thin ice, the majority of environmental-linked search and rescues are more nuanced. An example frequently discussed is that a river height will have increased dramatically over an afternoon and now a traveler has to go further to return home, causing them to run out of gasoline. As another example, with changing snow conditions

machines may be overheating more or hitting rocks leading to mechanical breakdowns, the leading cause of searches across the region [9, 10].

1.1 Search and rescue response in the Canadian Arctic

In Nunavut, many of the SAR volunteers are also Canadian Rangers, Civil Air Search and Rescue Association (CASARA) volunteers, Coast Guard Auxiliary volunteers, and active in local fire departments. While this makes for well-rounded and knowledgeable search teams, the response system is also dependent on a few very dedicated individuals.

While response by federal partners is rare, when the Royal Canadian Air Force (RCAF) does respond, it is usually from its southern air force bases. Canadian Air Force SAR bases are located in out of Greenwood, NS, Gander, NL, Trenton, ON, Winnipeg, MB, and Comox, BC. In terms of Arctic SAR resources, CC-130 Hercules and CH-149 Cormorants are usually used. Additionally, the CH-146 Griffon and CC-138 Twin Otter are used depending on availability, location of the incident, and performance demands. Canadian Coast Guard are usually only used when icebreakers are North during the summer.

With RCAF SAR assets not regularly stationed in Arctic communities beyond Yellowknife, private aircrafts are often hired out from mining companies or charter carriers, with a helicopter charter often running $10,000 per hour. We did not assess the coverage, cost, and response time of these *ad hoc* responses.

Data for aircraft performance was obtained from the RCAF website and reports [11] Costing projections were sourced from reports and newspaper articles [12, 13]. Variables used to calculate cost and response time were our best estimates given publicly accessible data (S1 Table). These figures should not be taken as true representations of performance, but simple the best estimates we were able to obtain.

S1 Table. RCAF aircraft specifications. To estimate response time and fuel cost for the Canadian Air Force to respond to communities across the Canadian Arctic, we used publicly available data on the most commonly used aircraft for the region. These values should be taken as our best estimate given available information.

	**CC-130 H**	**C-295**	**CH-149**	**CH-146**
**Hours flight time**	12.3 hours	7.3 hours	2.1	2.3
**Cruising speed (loaded with SAR gear)**	500 km/hr	481 km/hr	240 km/hr	185 km/hr
**Operating cost**	$30,792/hour	Unknown	$32,325/hour	$11,919/hour
**Fuel consumption (avg. trip with SAR load)**	4900lb/hour	1660lb/hour	Unknown	Unknown
**Range**	6480km	3520km	500km	420km

Additional assumptions used in our calculations included:

- Estimate of SAR rate is based on a ratio of 1.84 individuals per SAR tasking, derived from 2015 and 2016 data from the Government of Nunavut. In 2014, there were 543 SAR events above 55°N in Canada.
- A 45 minute decent/ascent and fuel time for the CC-295 – this is likely lower than average

- A 30 minute decent/ascent and fuel time for the CH-149 and CH-146 – this is likely lower than average
- Cost estimates include fuel, maintenance, flight crews, ground and operational support and amortized procurement
- Requirements for ETOPS (or RCAF mission risk assessments), diversion airport proximity, and surplus fuel were not incorporated into flight plans – these will further increase response time, considerably impacting helicopter and C-295 operations.
- We assumed the aircrew would time-out at 18 hours.

We chose to look at response times and costs for the designated SAR assets CC-130, CH149, and CH-146. While CC-138s are used, they are not in a designated SAR Wing. We briefly examine the response time of the expected new SAR fixed wing aircraft C-295, though more research is needed. The methods and variables used present a 'best case' scenario for the one-way response time. Further, we assumed that aircraft were available and serviceable from the closest CFB that is home to SAR fixed or rotor wing. For example, if an incident were to occur in Kuujjuaq, the CH-149 from CFB Gander offers the lowest response time – this is the time registered in the maps below. This assumption, however, is often not lived out in responses that are dictated by zone maps in which CFB Trenton covers much of Nunavut and the central Arctic; CFB Trenton is not the closest response base for fixed or rotor wing for any Arctic community.

It is important to consider that the estimated response times do not include and time searching, but simply arriving in the vicinity of a beacon or search. Further, it is important to recognize that under the current SAR response structure, if someone is in danger or experiencing a medical emergency then a SAR Technician would usually jump from the CC-130, stabilize the individual and make shelter, and wait for a helicopter to hoist or land. Herein, it is important to consider both the fixed wing and rotor wing response times and costs. Estimated fixed wing response time and costs are in the main paper, while helicopter response times and estimated costs can be found below (S1 and S2 Fig).

2. Study Method Details

2.1 Semi-structured interviews

We selected active harvesters and land-users by asking Hunters and Trappers Organizations for names and cross-validating the names with SAR committee members. Elders were identified by hamlet officials and prominent land-users. Emergency management officials interviewed included: Hamlet Senior Administrative Officer, Royal Canadian Mounted Police (RCMP) Officers, Community Health Representatives, nurses, SAR committee members, Canadian Rangers, and Hamlet Wildlife Officer. Interviews took place in both homes and offices after written consent was obtained. Sample size was determined by saturation. Interviews were conducted in English and Inuktitut with interpretation provided by locally hired research assistant. All interviews were audio recorded and conducted under Nunavut Research Institute License. In addition to semi-structured interviews, participants were asked for their feedback

after the flight sessions and a few days later. Feedback was provided orally and written down or recorded by the research team.

We then used thematic content analysis to selectively code for themes of emergency preparedness, emergency and disaster response, TK, technology and UAVs on the land, and landscape/hazard changes [4]. In our constant comparative analysis, we applied the following iterative steps: 1) Data within each category were reviewed to discern primary themes; 2) interviews were re-listened to, noting conversational tone and nuances; 3) data themes were mapped out within each category and links drawn.

2.2 UAV flight details

Flights were conducted under the rules and regulations established by Transport Canada and outlined in the Canadian Aviation Regulations, section 602.41. The research team went through UAV pilot training and was certified for use of airband radios. SAR volunteers practiced flying with the research team indoors and while on the land during hunting trips. Prior to assisting with piloting the UAVs, youth and SAR volunteers were taught the basics of flight mechanics, current operational rules and regulations, reviewed flight controls, and practiced flying on computer simulator. During all flights where a study participant helped fly a UAV, a trained UAV pilot was at their side. Initial flights were conducted indoors when possible. Due to safety and Transport Canada regulations, no flights were conducted with the operators inside a vehicle or building. Additional flights with high school students and SAR volunteers (over the age of 18) were conducted inside a school gymnasium.

We tested UAV mapping quality using both a crosshatch and cross-strip patterns flown by the pilot (not computer-generated tracks) while capturing photos every second. Flight speeds ranged from roughly 10 to 25km/hr. Images were taken with the gimbal positioned directly down. Images were uploaded to Pixel4Dmapper to create mesh orthostatic projections, digital elevation maps (DEMs), and 3D meshes.

3. Result and discussion details

S2 Table. Qualitative results summary. Qualitative semi-structured interviews demonstrated that a lack of volunteer resources and changing demands on the search and rescue system are impacting ability to affectively respond to emergencies.

**Emergency management and search and rescue capacity gaps and assets**	**Percent of participants that discussed theme**	**Central implication**
System is highly dependent on volunteers	56% (n=10)	High volunteer burnout and lack of multilevel support and collaboration
Training and resource deficits	44% (n=8)	Diminished ability to respond to complex emergencies
Increasing and more complex search and rescues	44% (n=8)	System is increasingly struggling to adequately and safely meet needs
Volunteers have strong levels of local and Indigenous Knowledge	44% (n=8)	Improved ability to navigate landscape and knowledge of local values and needs
Rapidly shifting social and economic contexts	61% (n=11)	Indigenous knowledge levels and land skills are changing and there is greater available of

		technology (GPS or satellite phones) for some individuals

Successful flights were conducted down to temperatures of -7°C, and up to +8°C. Maximum wind speed operated in was 20km/hr. Cloud ceilings were always greater than 4000’ above ground level and visibility was always greater than 5nm (S3 Fig). UAV systems worked well in all conditions, although 20km wind speeds were felt to be the maximum operators were comfortable operating in. No differences were noticed with UAV performance based on the temperature range tested.

This 3D mesh clearly depicted vehicles, floating ice, and rocks larger than 60cm diameter. The second-best quality 3D mesh was produced with the track crawl flight pattern at 23m. This mesh depicted cabins, roads, and large snowbanks, though with less discernable quality. The 3D mesh quality from 38m was beneficial only for assessing large topographical features (S4 Fig).

4. References

1. Clark DG, Ford JD. Emergency response in a rapidly changing Arctic. Cmaj Canadian Medical Association Journal 2017;189(4):E135-E136.
2. Durkalec A, Furgal C, Skinner MW, Sheldon T. Investigating Environmental Determinants of Injury and Trauma in the Canadian North. International Journal of Environmental Research and Public Health 2014;11(2):1536-1548.
3. Young S, Tabish T, Pollock N, Young T. Backcountry Travel Emergencies in Arctic Canada: A Pilot Study in Public Health Surveillance. International Journal of Environmental Research and Public Health 2016;13(3):276.
4. Clark DG. Vulnerability to Injury: assessing biophysical and social determinants of land-user injuries in Nunavut, Canada: McGill University; 2016.

5. Aporta C. Routes, trails and tracks: Trail breaking among the Inuit of Igloolik. Inuit Studies 2004;28(2):9-38.
6. Pearce T, Ford J, Cunsolo Willox A, Smit B. Inuit Traditional Ecological Knowledge (TEK), Subsistence Hunting and Adaptation to Climate Change in the Canadian Arctic. Arctic 2015;68(2):233-245.
7. Ford JD, McDowell G, Shirley J, Pitre M, Siewierski R, Gough W, et al. The Dynamic Multiscale Nature of Climate Change Vulnerability: An Inuit Harvesting Example. Annals of the Association of American Geographers 2013;103(5):1193-1211.
8. Gearheard S, Pocernich M, Stewart R, Sanguya J, Huntington HP. Linking Inuit knowledge and meteorological station observations to understand changing wind patterns at Clyde River, Nunavut. Climatic Change 2010;100(2):267-294.
9. Clark DG, Ford J, D.,, Berrang-Ford L, Pearce T, Gough WA, Kowal S. The role of environmental factors in search and rescue incidents in Nunavut, Canada. Public Health 2016(137):44-49.
10. Durkalec A, Furgal C, Skinner MW, Sheldon T. Climate change influences on environment as a determinant of Indigenous health: Relationships to place, sea ice, and health in an Inuit community. Social Science & Medicine 2015;136:17-26.
11. Department of National Defense. Evaluation of the DND/CAF Contribution to the National Search and Rescue Program. Ottawa: Government of Canada; 2015.
12. Kosucu K. Estimating the Operating and Support Cost Difference Among TUAF C-130, C-130B and USAF C-130J Aircraft: AIR FORCE INST OF TECH WRIGHT-PATTERSONAFB OH SCHOOL OF SYSTEMS AND LOGISTICS; 2002.
13. Humphreys A. Canadian military spent $2.7 million to rescue wealthy tourists off of breakaway ice floe. National Post 2013.
14. Koster R, Baccar K, Lemelin RH. Moving from research ON, to research WITH and FOR Indigenous communities: A critical reflection on community-based participatory research. The Canadian Geographer/Le Géographe canadien 2012;56(2):195-210.
