# Supplementary figures and images for: What role can unmanned aerial vehicles play in emergency response in the Arctic: A case study from Canada
Source: PLoS One. 2018 Dec 18;13(12):e0205299. doi: 10.1371/journal.pone.0205299 (PMC6298648; doi:10.1371/journal.pone.0205299)

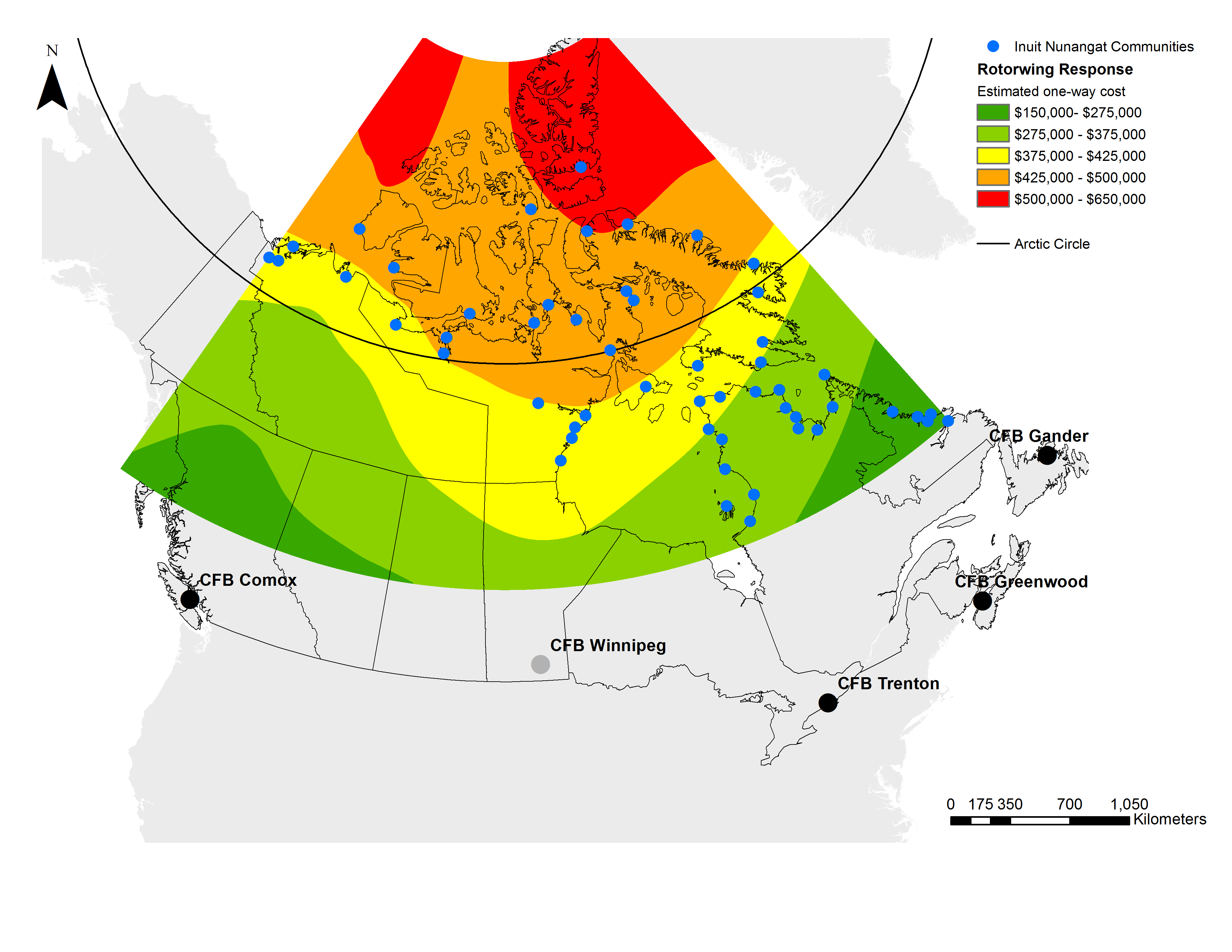

Supplement: S1 Fig — Estimated response cost for CH-149 and CH-146 to respond to SAR incidents across the Canadian Arctic. Costs are estimated for the aircraft that could arrive at the location quickest from respective bases (CFB Comox, CFB Trenton, CFB Greenwood, and CFB Gander). Basemap shapefiles are modified and republished from Government of Canada under a CC BY license, with permission from Natural Resources Canada and Crown-Indigenous Relations, original copyright 2018. Contains information licensed under the Open Government License–Canada (11,12). (TIF) [file pone.0205299.s002.tif]

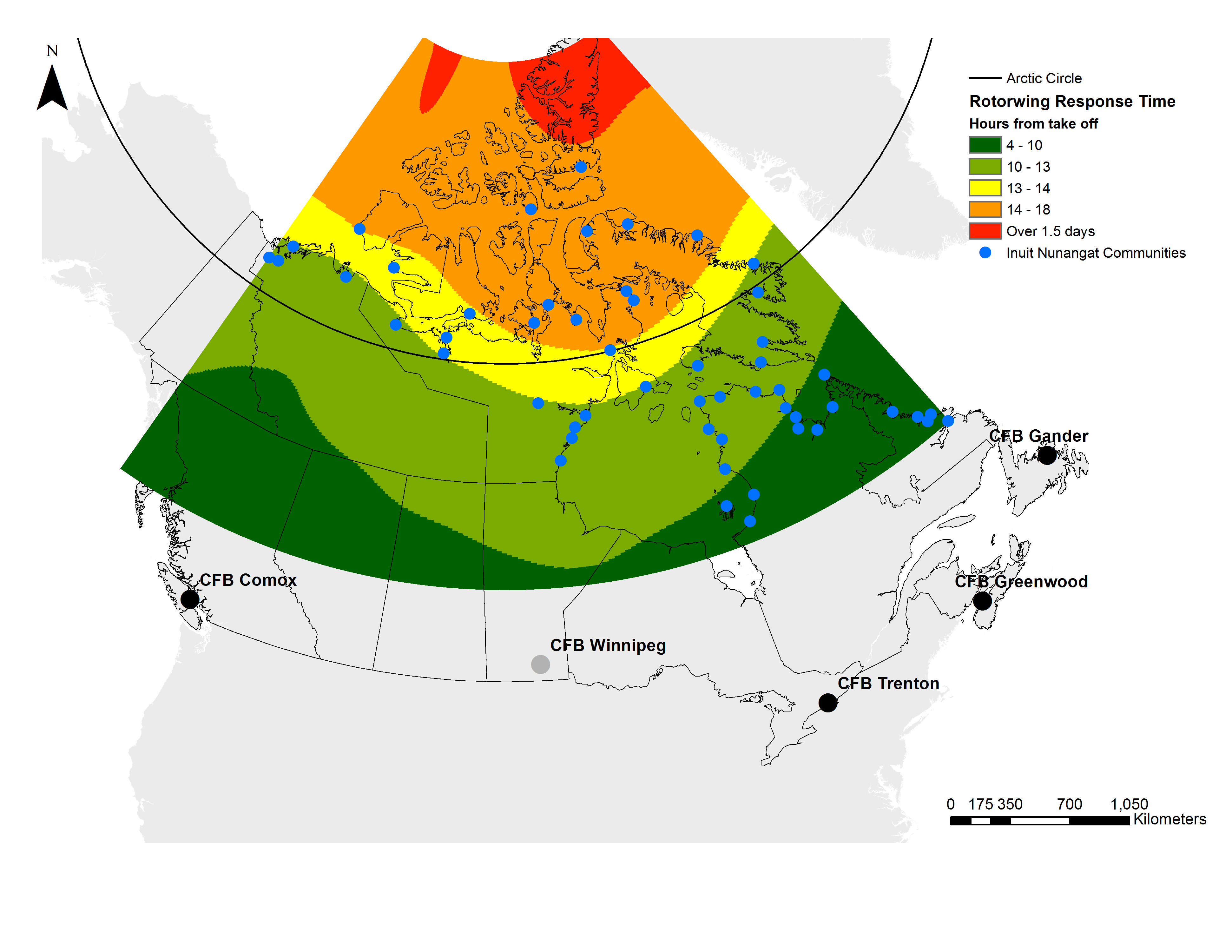

Supplement: S2 Fig — Estimated response time from takeoff to incident location for CH-149 and CH-146 to respond to SAR incidents across the Canadian Arctic. Times are estimated for the aircraft that could arrive at the location quickest from respective bases (CFB Comox, CFB Trenton, CFB Greenwood, and CFB Gander). Basemap shapefiles are modified and republished from Government of Canada under a CC BY license, with permission from Natural Resources Canada and Crown-Indigenous Relations, original copyright 2018. Contains information licensed under the Open Government License–Canada [11,12]. (TIF) [file pone.0205299.s003.tif]

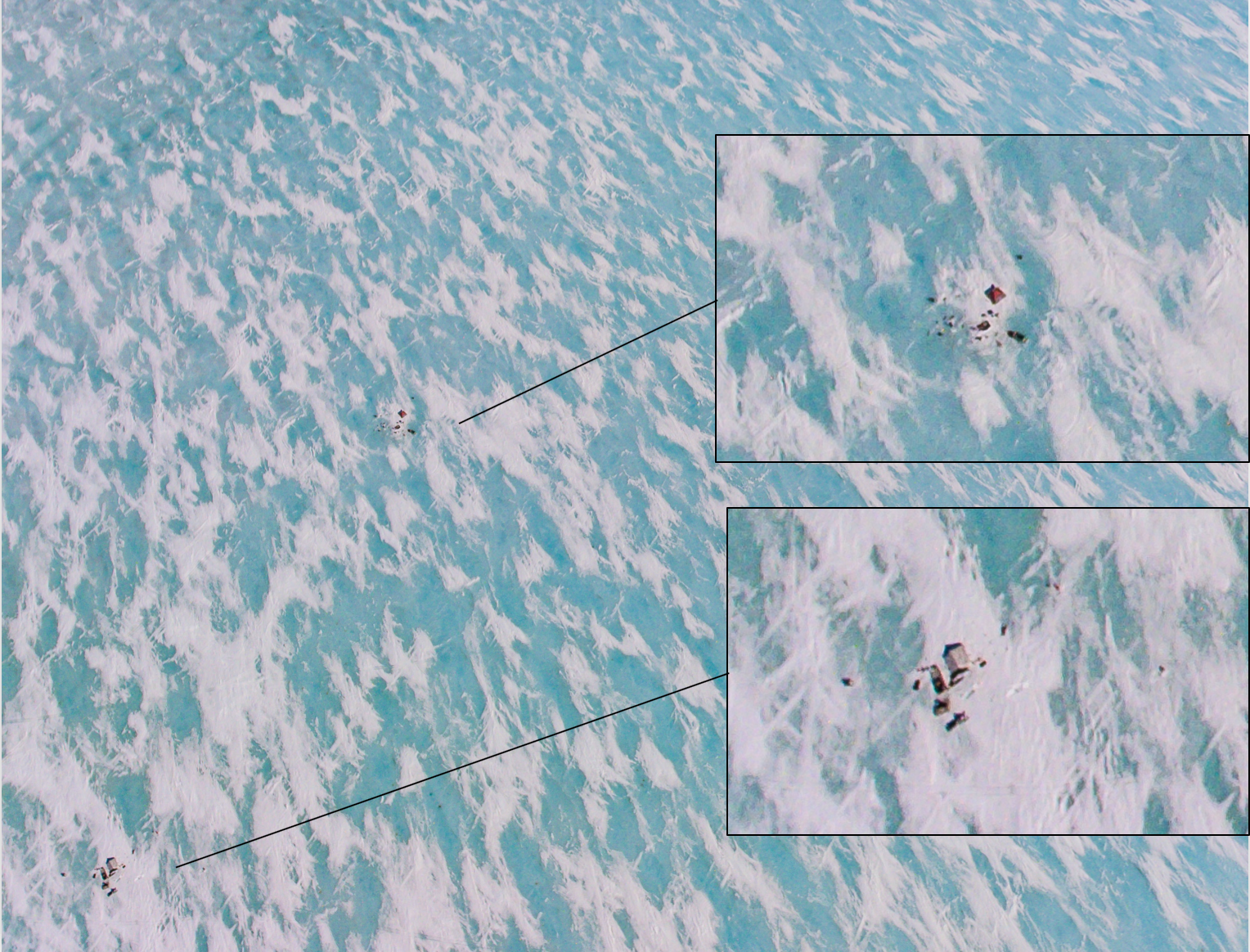

Supplement: S3 Fig — This photo captured at roughly 300’ above ground level, depicts two groups ice fishing on the Hudson Bay near Arviat Nunavut. In high contrast environments, such as melting sea ice, targets can be seen at higher altitudes with large screens. However, participants and researchers found it difficult to spot targets in lower contrast environments, such as bare ground, and while using smaller screens for real-time image feeds. Photo taken by Dylan G. Clark, with approval to distribute under the CC BY license. (TIF) [file pone.0205299.s004.tif]

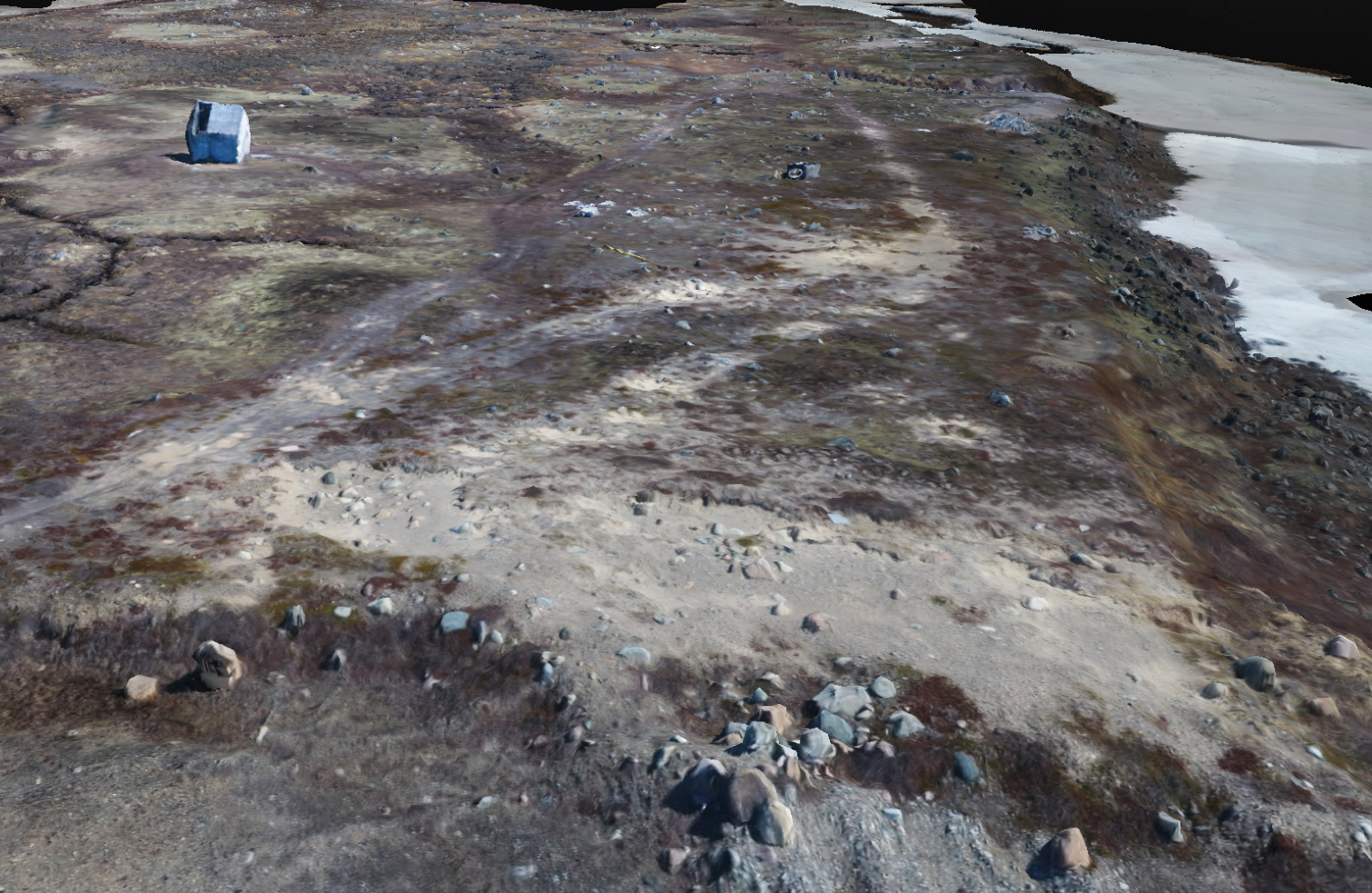

Supplement: S4 Fig — This photo captures the power of the three-dimension meshing produced using UAV images taken at approximately 75’ above ground level. In this photo you can see a wooden cabin, ATV trail, shore line dropping off to the right of the photo, and the visible sand and rock pits in the foreground. Photo generated by Dylan G. Clark, with approval to distribute under the CC BY license. (TIF) [file pone.0205299.s005.tif]
